# Activation of orexin-2 receptors in the Kӧlliker-Fuse nucleus of anesthetized mice leads to transient slowing of respiratory rate

**DOI:** 10.3389/fphys.2022.977569

**Published:** 2022-11-02

**Authors:** Adrienn G. Varga, Jessica R. Whitaker-Fornek, Sebastian N. Maletz, Erica S. Levitt

**Affiliations:** ^1^ Department of Pharmacology and Therapeutics, University of Florida, Gainesville, FL, United States; ^2^ Breathing Research and Therapeutics Center, University of Florida, Gainesville, FL, United States

**Keywords:** Kӧlliker-Fuse nucleus, orexin, orexin-B peptide, MDK 5220, respiration, microinjection, bradypnea

## Abstract

Orexins are neuropeptides originating from the hypothalamus that serve broad physiological roles, including the regulation of autonomic function, sleep-wake states, arousal and breathing. Lack of orexins may lead to narcolepsy and sleep disordered breathing. Orexinergic hypothalamic neurons send fibers to Kӧlliker-Fuse (KF) neurons that directly project to the rostroventral respiratory group, and phrenic and hypoglossal motor neurons. These connections indicate a potential role of orexin-modulated KF neurons in functionally linking the control of wakefulness/arousal and respiration. In a reduced preparation of juvenile rats Orexin B microinjected into the KF led to a transient increase in respiratory rate and hypoglossal output, however Orexin B modulation of the KF in intact preparations has not been explored. Here, we performed microinjections of the Orexin B mouse peptide and the synthetic Orexin 2 receptor agonist, MDK 5220, in the KF of spontaneously breathing, isoflurane anesthetized wild type mice. Microinjection of Orexin-2 receptor agonists into the KF led to transient slowing of respiratory rate, which was more exaggerated in response to Orexin-B than MDK 5220 injections. Our data suggest that Orexin B signaling in the KF may contribute to arousal-mediated respiratory responses.

## Introduction

Orexins (ORX) or hypocretins are neuropeptides expressed in two splice variants (ORX-A and ORX-B), that are exclusively produced by hypothalamic neurons in the brain (Wang et al., 2018). ORXs stimulate target neurons by binding to G-protein coupled receptors with two identified isoforms, Orexin-1 receptor (OX1R) and Orexin-2 receptor (OX2R). ORX-A binds to both OX1R and OX2R with equal affinity, whereas ORX-B binds with higher affinity to OX2R (Sakurai et al., 1998).

Due to the widespread expression of ORX receptors throughout the brain, the ORX system interacts with a wide array of specialized circuits and regulates diverse behaviors, including sleep-wake control, energy homeostasis, feeding, and reward processing (Peyron et al., 1998; Harris and Aston-Jones, 2006; Sakurai, 2007). Additionally, a growing body of literature supports a role for ORX neurons in the control of breathing (Deng et al., 2007; Kuwaki, 2008; Kuwaki et al., 2008; Williams and Burdakov, 2008), likely *via* direct projections to respiratory nuclei, such as the parabrachial and Kӧlliker-Fuse nuclei (KF; (Peyron et al., 1998; Yokota et al., 2016), pre-Bӧtzinger complex (Young et al., 2005), retrotrapezoid nucleus (Dias et al., 2009; Lazarenko et al., 2011), nucleus of the solitary tract (Nambu et al., 1999), and trigeminal and hypoglossal nuclei (Fung et al., 2001; Young et al., 2005). While ORXs do not appear to contribute to the control of basal respiration, their importance is evident in arousal-state dependent changes in breathing (Kuwaki, 2008; Kuwaki et al., 2008; Williams and Burdakov, 2008; Nattie and Li, 2012). This is supported by data from ORX receptor knock-out mice, which show significantly attenuated hypercapnic ventilatory responses during wakefulness and are more prone to spontaneous apneas during sleep compared to wild type mice (Nakamura et al., 2007; Williams and Burdakov, 2008).

The parabrachial nuclei, including the KF, receive projections that carry chemosensory information (hypercapnia and hypoxia) from the medulla, as well as mechanosensory signals associated with upper airway pressures during apneas (Kaur and Saper, 2019). Additionally, parabrachial and KF neurons are known to display increased activity during wakefulness (Lydic and Orem, 1979; Sieck and Harper, 1980). Thus, based on these data, we predict that ORX-modulated KF neurons participate in the arousal-dependent control of respiration.

The KF is essential for maintenance of upper airway patency (Dutschmann and Herbert, 2006), which is increased during wakefulness compared to sleep (Remmers et al., 1978; Oliven et al., 2020). ORX-B microinjected into the KF of rats produced transient increases in respiratory rate that also coincided with an increase in hypoglossal motor output, linking ORX modulation of KF to increased upper airway patency (Dutschmann et al., 2007). In addition to providing initial insight into the possible role of ORX signaling in the KF, this study also supports anatomical evidence of OX2R in the KF in the rat (Marcus et al., 2001). Nevertheless, since these experiments utilized the *in situ* working heart-brainstem preparation of juvenile rats, there remains a gap in our knowledge regarding the effects of orexinergic signaling in the KF in intact adult animals.

Despite significant progress in our understanding of the role of ORX-B in respiratory control, the relationship between local effects of ORX-B signaling in distinct respiratory control centers and global changes in breathing behavior are still not clear. Here, we performed microinjections of the ORX-B mouse peptide and the synthetic OX2R agonist, MDK-5220, in the KF of spontaneously breathing, isoflurane anesthetized, adult wild type mice. Microinjection of both OX2R agonists into the KF led to transient decreases in breathing frequency, and a pattern reminiscent of breath hold apneas that occur following stimulation of the KF and are associated with increased activity of upper airway motor neurons (Chamberlin and Saper, 1994; Dutschmann and Herbert, 2006). These findings suggest that ORX-B signaling in the KF of fully intact, adult mice may be important for the induction of post-arousal apneas or bradypnea, and other arousal-mediated respiratory responses.

## Methods

### Animals

Male and female wild type C57/BL6J mice (2–6 months old) were used for all experiments. Mice were bred and maintained at the University of Florida animal facility. Mice were group-housed in standard sized plastic cages and kept on a 12 h light–dark cycle, with water and food available *ad libitum*. Experiments were performed during the light cycle. All procedures were conducted in accordance with the National Institutes of Health guidelines and with approval from the Institutional Animal Care and Use Committee of the University of Florida.

### Respiratory inductance plethysmography

Mice were anesthetized with isoflurane (1.5–2% in O_2_; Zoetis, Parsippany-Troy Hills, NJ, United States) and mounted in a stereotaxic frame (Kopf Instruments, Tujunga, CA, United States) where anesthesia was maintained through a nose cone at a flow rate of 0.8 ml/min. Respiratory activity was measured with a pulse transducer (MP100, AD Instruments, Colorado Springs, CO, United States) fixed against the abdomen of mice with a Velcro strap. This allowed us to measure respiratory rate and relative changes in amplitude. Recordings were digitized with PowerLab (ADInstruments, LabChart 8 software), and respiratory data were processed offline with LabChart 8 software and exported into Microsoft Excel.

### Stereotaxic microinjections in anesthetized mice

**FIGURE 1:**
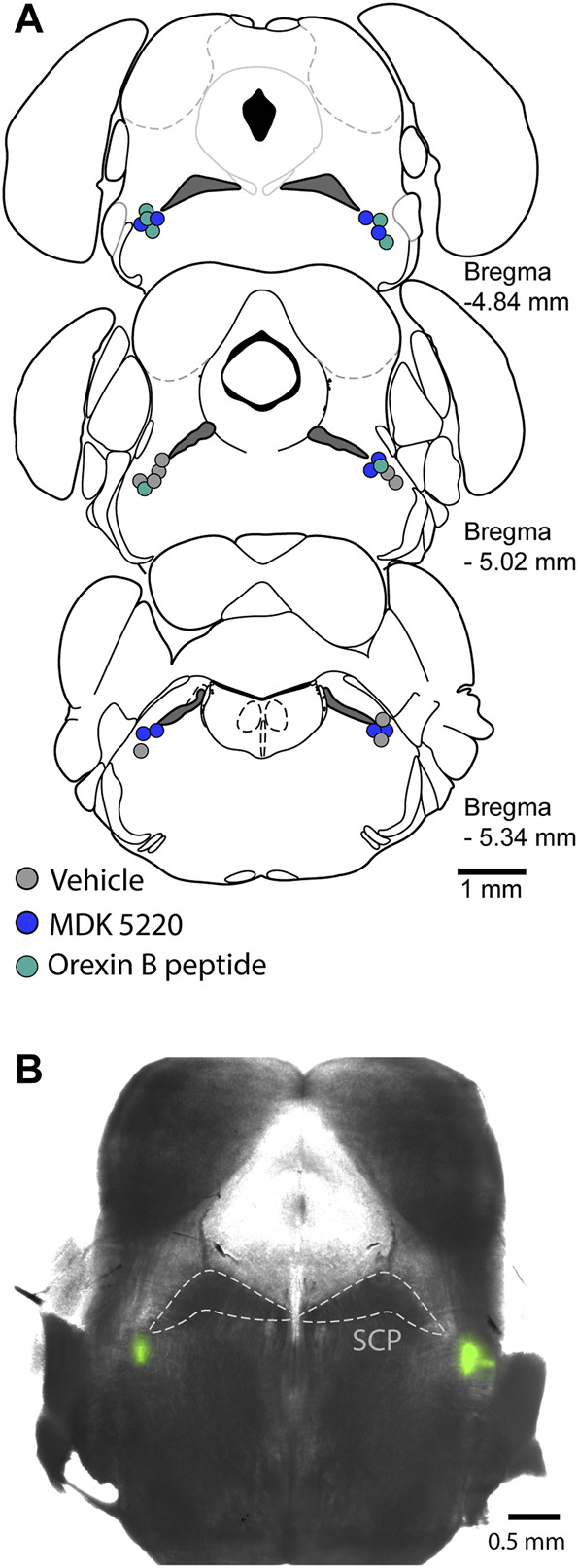
Microinjections were targeted to the KF. **(A)** Schematic maps of injection sites along the rostral-caudal extent of the KF. Each colored circle represents the site of a complete experiment, with three consecutive 50 nl and two consecutive 100 nl injections of either vehicle, MDK 5220 or Orexin B peptide. **(B)** Representative micrograph of a coronal brain slice containing bilateral KF microinjections marked with FluoSpheres. SCP, superior cerebellar peduncle.

After mice were positioned in the stereotaxic frame and their respiratory rate stabilized at ∼30 breaths/min, the dorsal skull was exposed and levelled horizontally between bregma and lambda. A small craniotomy was made to access the KF bilaterally (y = −5 mm, x = ±1.7 mm, from bregma). Each mouse received a single treatment *via* the following procedure. A glass micropipette filled with either saline, saline + 1% DMSO, MDK 5220 (1 mM in saline) or Orexin B peptide (ORX-B, 10 µM in saline) mixed with 2% FluoSpheres (0.04 µm, 505/515 nm, Invitrogen, Eugene OR, United States; for visualization of the injection sites) was slowly lowered into the KF (z = −3.85 mm). Mice received 5 consecutive microinjections (3 × 50 nl and 2 × 100 nl, at a rate of 50 nl/s, with >2 min inter-injection interval) in each hemisphere controlled by a Nanoject III (Drummond Scientific Company, Broomall, PA, United States). Following the final injection, the pipet was slowly retracted, mice were deeply anesthetized, then decapitated, and the brains were removed and freshly sliced or fixed in 10% formalin (>24 h). For histological identification of the injection sites, 100 µm coronal sections were cut in PBS on a Leica VT 1200S vibratome (Leica Biosystems, Buffalo Grove, IL, United States). Injection sites were marked with FluoSpheres (Figure 1), and all experiments where the marked site was outside of the KF region were analyzed as a separate group for off-target effects. Injection sites were visualized in free-floating sections, and images were collected on a multizoom microscope (Nikon AZ100) with a ×1 objective (N.A. 0.1) and individually processed in Fiji (Schindelin et al., 2012).

### Drugs

Orexin B mouse peptide (cat # 1457, batch 6A) was from Tocris (Ellisville, MO, United States), and a 1 mM stock solution (in ddH2O) was diluted to 10 µM in saline on experiment days. The OX2R agonist, MDK 5220 (cat #A15982) (also known as YNT-185 (Irukayama-Tomobe et al., 2017; Toyama et al., 2018)), was from AdooQ Bioscience (Irvine, CA, United States). The 10 mM stock solution (in DMSO) was freshly diluted to 1 mM in saline on experiment days.

### Data analysis

All statistical analyses were performed in GraphPad Prism 9 (San Diego, CA, United States). All error bars represent SEM unless otherwise stated. We did not find any influence of sex on our measured variables in the vehicle groups and the MDK 5220 injected group, thus data for males and females were combined before statistical analyses. The ORX-B injected group only included males. Data were tested for normality with the Kolmogorov–Smirnov test. Since our data were normally distributed, comparisons between two groups were made using Student’s two-tailed *t*-test. Comparisons between three or more groups were made using one-way ANOVA or two-way ANOVA followed by Tukey’s *post hoc* test. Statistical significance is defined as *p* < 0.05.

## Results

**FIGURE 2:**
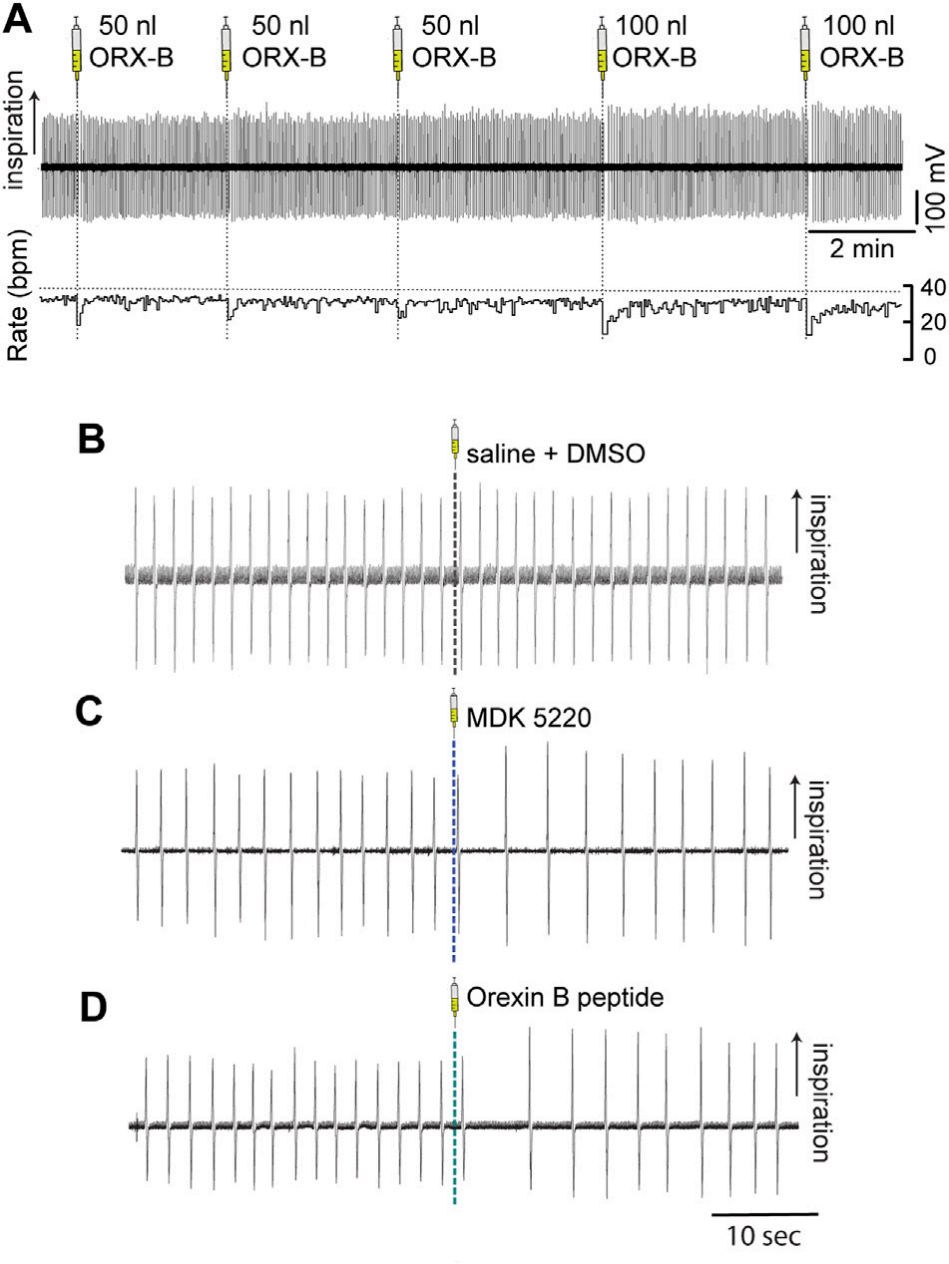
Microinjections of OX2R agonists into the KF transiently slowed breathing in anesthetized adult mice. Respiration was monitored with induction plethysmography. In this representative example trace **(A)** Orexin B peptide was injected into the KF in the left hemisphere in multiple consecutive injections (three 50 nl and two 100 nl Orexin B peptide injections) in a continuous experiment. Orexin B peptide injections led to volume-dependent reductions in respiratory rate (bottom trace; rate measured in breaths per minute [bpm]). Representative example traces of breathing immediately before and after 100 nl microinjections of either vehicle **(B)**, MDK 5220 **(C)**, or Orexin B peptide **(D)** into the KF.

The main goal of this study was to determine the effect of the OX2R agonists, MDK 5220 (*n* = 10 mice) and ORX-B mouse peptide (*n* = 7 mice), on respiration following local microinjections into the KF of adult, vagal-intact, anesthetized, wild type mice (see injection sites in Figure 1). We focused our analyses on changes in respiratory rate, inter-breath interval and amplitude. Since both agonists appeared to have a transient effect on breathing (Figure 2), we analyzed changes in respiration during the first 6 breaths post-injection. We compared the agonist effect to a control group, which consisted of animals microinjected in the KF with saline (*n* = 4 mice) or saline + 1% DMSO (*n* = 5 mice). Data from these control groups were similar (mean frequency ± SEM, saline: 27.86 ± 1.65 breaths/min vs. saline + DMSO: 29.18 ± 0.86 breaths/min, *p* = 0.53 with two-tailed unpaired *t*-test), so they were pooled and are henceforth referred to as the “vehicle” group. Additionally, since the level of anesthesia was controlled by the experimenter, and also potentially affected by individual variability in the animals’ susceptibility to isoflurane, we also performed within-subject comparisons, where breathing immediately preceding an injection (6 breaths pre-injection) was compared to or normalized to breathing immediately following the injection (6 breaths post-injection).

**FIGURE 3:**
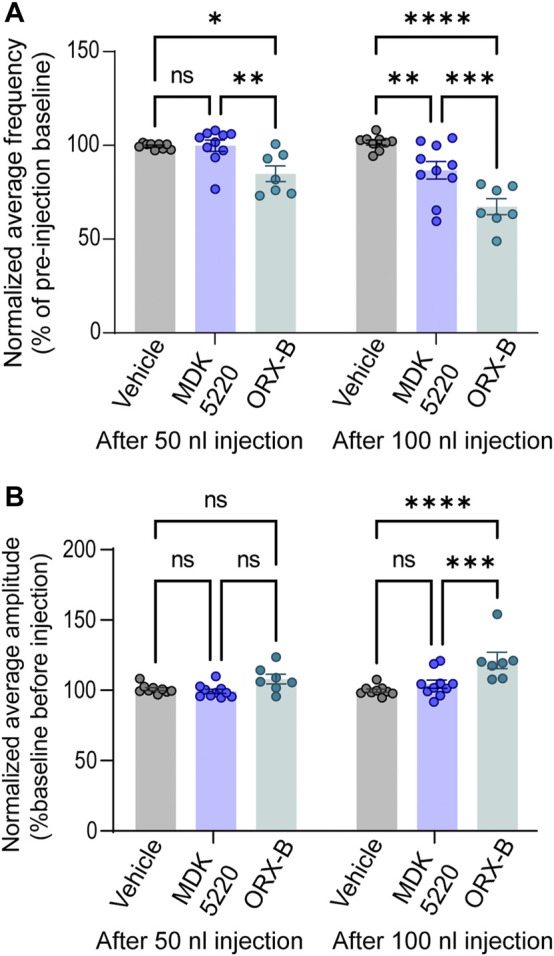
MDK 5220 in the KF decreased respiratory frequency, while ORX-B peptide affected both frequency and amplitude. **(A)** ORX-**B** (50 nl) was sufficient to decrease breathing frequency compared to pre-injection baseline, whereas MDK 5220 (100 nl) was needed to evoke a similar effect. Regardless of injection volume, vehicle injections did not change breathing frequency. **(B)** Only 100 nl ORX-B peptide injections lead to significant changes in amplitude. Each data point represents the normalized mean frequency **(A)** and amplitude **(B)** per injection site (mean of three consecutive 50 nl injections in the graphs on the left, and mean of two consecutive 100 nl injections in the graphs on the right). Bars and error bars are normalized group mean ± SEM. *p < 0.05, **p < 0.01, ***p < 0.001, ****p < 0.0001 with two-way ANOVA and Tukey’s *post hoc* tests.

Vehicle injections of any bolus volume did not have a substantial effect on the average frequency when normalized to baseline breathing preceding the injection (Figures 2B, 3A; normalized mean frequency ± SEM, vehicle [50 nl] 99.63 ± 0.51%, vehicle [100 nl] 101.49 ± 1.32% compared to baseline). On the other hand, ORX-B peptide [50 nl] injections significantly decreased the normalized average breathing frequency compared to vehicle injections (Figures 2A, 3A; mean frequency ± SEM, ORX-B [50 nl] 84.86 ± 4.22%, *p* = 0.01 compared to vehicle, with two-way ANOVA and Tukey’s *post hoc* test). When we increased the injection volume to 100 nl, both the agonist MDK 5220 and ORX-B peptide significantly decreased the average normalized breathing frequency compared to the vehicle group (Figures 2A,C, D, 3A; MDK 5220 86.71 ± 4.69% of baseline, MDK 5220 vs. vehicle *p* = 0.005; ORX-B 67.31 ± 4.19% of baseline, ORX-B vs. vehicle *p* < 0.0001; with two-way ANOVA and Tukey’s *post hoc* test). These data indicate that activation of KF OX2Rs by either agonist leads to a substantial decrease in respiratory rate when applied under anesthesia.

In contrast with respiratory rate, out of all conditions, only 100 nl ORX-B peptide injections led to a significant increase in amplitude (Figures 2A,D, 3B; normalized mean amplitude ± SEM [100 nl] 120.56 ± 5.87% compared to baseline; [100 nl] vehicle vs. ORX-B *p < 0.0001*; [100 nl] MDK 5220 vs. ORX-B *p = 0.0004*, with two-way ANOVA and Tukey’s *post hoc* test).

**FIGURE 4:**
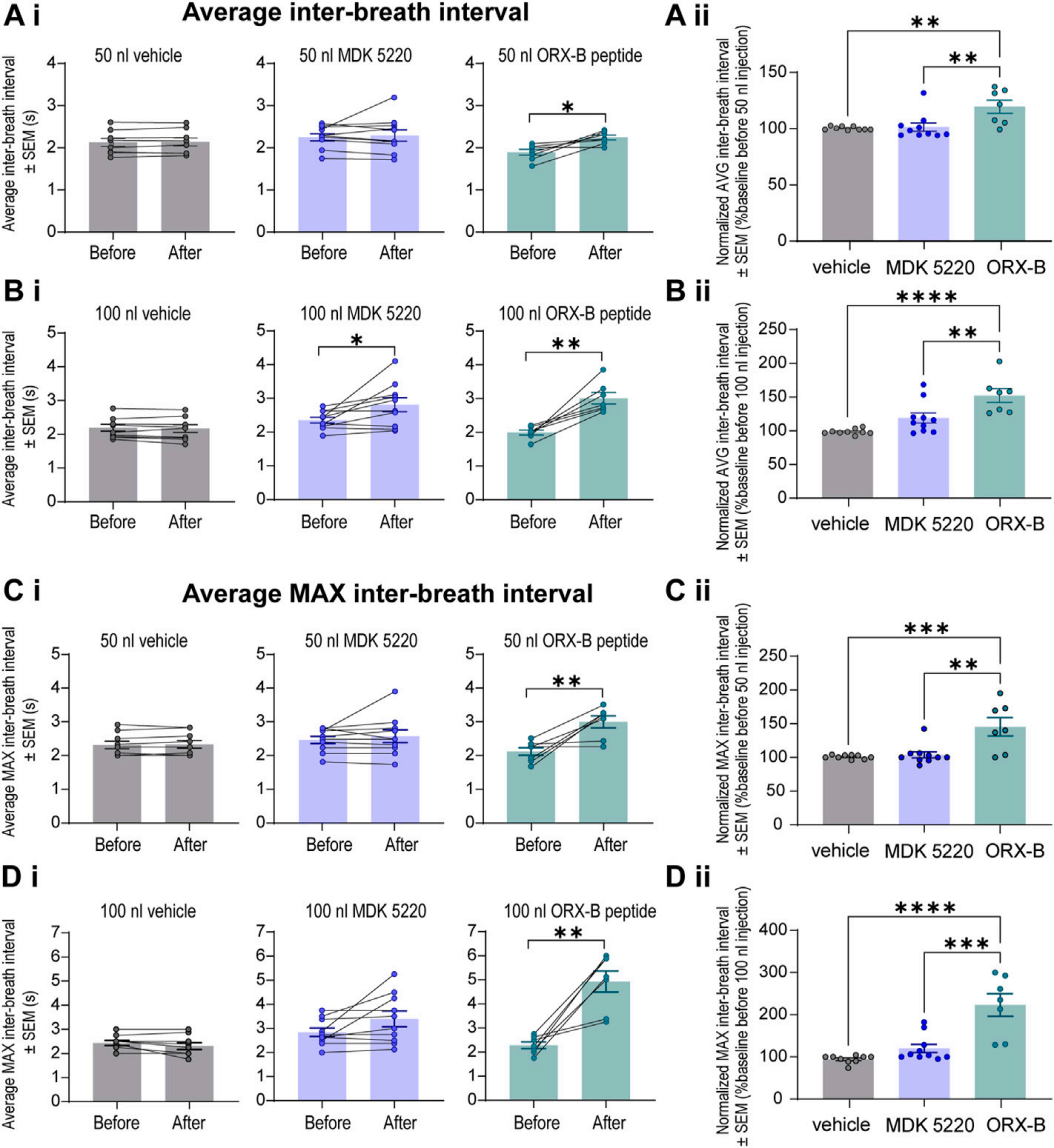
Local ORX-B application to the KF triggered longer mean and maximum inter-breath intervals while the effects of MDK 5220 were volume-dependent. **(Ai)**. Mean inter-breath interval (IBI) for baseline breathing (before) and post-injection (after) are plotted by injection type (columns) for 50 nl injections. Mean IBI normalized to baseline breathing is shown in **(Aii)**. Mean and normalized mean IBI for 100 nl injections are similarly plotted in **(Bi,Bii)**. Mean maximum IBI for baseline breathing before and following 50 nl and 100 nl microinjections are plotted in **(Ci**,**Di)**. Normalized mean maximum IBI are plotted for 50 nl and 100 nl microinjections in **(Cii,Dii)**. Each data point represents the average IBI or average maximum IBI per injection site (the average of three consecutive 50 nl injections, and the average of two consecutive 100 nl injections). Bars and error bars are group mean ± SEM. *p < 0.05, **p < 0.01, ***p < 0.001, ****p < 0.0001, with paired two-tailed *t*-test **(Ai–Di)** or one-way ANOVA and Tukey’s *post hoc* test **(Aii–Dii)**.

The observed decreases in breathing frequency correspond with increases in the duration of inter-breath intervals (IBI, Figure 4). Mice that received the ORX-B peptide (50 nl) had significantly longer IBIs, that were 19.6 ± 5.85% longer than before drug injection (Figure 4Ai, mean IBI before 1.89 ± 0.07s vs. after 2.25 ± 0.06s, *p* = 0.01, paired two-tailed *t*-test). Meanwhile, although the effect was still transient, mice that received the 100 nl MDK 5220 or 100 nl ORX-B peptide injections both displayed significantly longer IBIs, with the ORX-B peptide having a stronger effect (Figure 4Bi, MDK 5220 mean IBI before 2.36 ± 0.09 s vs. after 2.81 ± 0.20 s, *p* = 0.03; ORX-B mean IBI before 1.99 ± 0.07 s vs. after 3.01 ± 0.17 s, *p* = 0.002; paired two-tailed *t*-test). Following 100 nl MDK 5220 injections the average interval between breaths increased by 19.1 ± 7.54%, while 100 nl ORX-B peptide injections caused a 52.3 ± 10.30% increase in average IBI duration compared to baseline durations prior to the injections (Figure 4Bii).

To better illustrate the magnitude of the longest pause between breaths following OX2R receptor activation, average maximum IBIs were quantified (Figures 4C,D). After the 50 nl ORX-B injection the average maximum IBI was 45 ± 13.54% longer than during the baseline period before the injections (Figures 4Ci,Cii; mean max IBI before 2.11 ± 0.12 s vs. after 2.99 ± 0.18 s; *p* = 0.009, paired two-tailed *t*-test). There was an even more substantial 123 ± 26.76% increase after 100 nl injections when compared to the baseline breathing period (Figures 4Di,Dii; mean max IBI before 2.29 ± 0.14s vs. after 4.93 ± 0.44s; *p* = 0.002, paired two-tailed *t*-test). As also shown in Figure 2, it appeared visually that following the 100 nl injections there was a trend for the agonist MDK 5220 in lengthening the maximum inter-breath interval as well, however these results are not statistically significant (Figures 4Di,Dii; 120.1 ± 9.89% of baseline, mean max IBI before 2.84 ± 0.17 s vs. avg after 3.40 ± 0.33 s after, *p* = 0.069, paired two-tailed *t*-test). Thus, the ORX-B mouse peptide has a strong, volume-dependent, influence on increasing the average maximal inter-breath intervals compared to baseline values, leading to longer pauses between breaths after local injection in the KF.

Importantly, experiments with off target injection sites (medial parabrachial nucleus, lateral parabrachial nucleus, middle cerebellar peduncle, and trigeminal nucleus) resulted in no significant changes in amplitude, frequency, and inter-breath interval (within subject comparisons with paired *t*-test, *p* > 0.05; group comparisons with the vehicle group with two-way ANOVA and Tukey’s post hoc test, *p* > 0.05), even though some of these areas also express OX2Rs (Greco and Shiromani, 2001; Marcus et al., 2001). These experiments provide additional support for KF neurons mediating the slowing effects of OX2R agonists on breathing.

## Discussion

The results of this study show that activation of OX2R agonists in the KF has a significant impact on breathing in adult, vagal-intact, anesthetized, wild type mice. Microinjections of both the synthetic agonist MDK 5220 and ORX-B mouse peptide into the KF acutely decreased breathing frequency (Figure 3A). ORX-B also significantly increased the amplitude (Figure 3B), and average and maximum inter-breath intervals (Figure 4). Investigating the role of orexinergic signaling in the KF is critical for beginning to understand the hypothalamic-brainstem circuits that allow respiration to be adjusted according to arousal or sleep-wake states.

OX2R activation in the KF of anesthetized, adult mice may contribute to temporary breath holds or bradypnea, by engaging pontine inspiratory off-switch circuitry (Lumsden, 1923; Cohen, 1971), similar to the effects of excitation by glutamate (Chamberlin and Saper, 1994). A comprehensive study of the KF/lateral parabrachial area in anesthetized rats revealed that glutamate microinjections into the lateral and ventral KF causes brief apneas and resets the respiratory rhythm (Chamberlin and Saper, 1994). Since OX2R activation depolarizes neurons by closing potassium channels (Horvath et al., 1999; Ivanov and Aston-Jones, 2000), local OX2R activation in the KF may similarly excite neurons that participate in the respiratory control circuit that terminate inspiratory effort. Stimulation of the KF also increases the activity of motor neurons that control the upper airways (Dutschmann and Herbert, 2006). Upper airway patency is decreased during sleep and impaired during obstructive sleep apnea (Remmers et al., 1978; Oliven et al., 2020). Orexinergic signaling in the KF during wakefulness could provide an excitatory tone that helps maintain tonic drive to motor neurons that control the upper airways and support patency.

In this study we found that the synthetic OX2R agonist MDK 5220 and the endogenous mouse ORX-B peptide injected in the KF changed respiration to different degrees. Despite using a higher concentration of the synthetic agonist (1 mM MDK 5220), the ORX-B peptide at 10 µM led to significant changes in amplitude, frequency and inter-breath interval even when less volume was injected (Figures 2–4). While both agonists have similar potency and binding affinity to OX2R (Sakurai et al., 1998; Irukayama-Tomobe et al., 2017), the OXR-B peptide is less selective and may also bind to OX1R (Sakurai et al., 1998; Kilduff and Peyron, 2000), which may explain the more substantial changes in respiratory pattern following peptide injections. OX1R expression has been described in the KF and parabrachial region in rodents (Marcus et al., 2001; Varga et al., 2020), and the KF in humans (Lavezzi et al., 2016). Histological examination of the KF of infants who died of sudden infant death syndrome (SIDS) showed significantly reduced levels of OX1R expression compared to non-SIDS cases (Lavezzi et al., 2016). Since SIDS can result from failure to arouse from sleep in response to a potentially life-threatening stressor, deficient OX1R expressing KF neurons may impair breathing during the transition period from sleep to wakefulness. Thus, it is likely that in our experiments ORX-B peptides binding to OX1R, in addition to OX2R, induced exaggerated changes in breathing compared to the synthetic agonist MDK 5220.

### Limitations

It is necessary to consider the experimental preparation and conditions when interpreting the respiratory consequences of exciting KF neurons *via* ORX-B agonist microinjection. In contrast to our findings, local ORX-B delivery to the KF in the *in situ* working heart-brainstem preparation (WHBP) of juvenile rats increased phrenic and hypoglossal nerve burst frequency (Dutschmann et al., 2007). The WHBP contains the ponto-medullary respiratory network and it produces a 3-phase, respiratory motor output (Paton et al., 2022). One of the advantages of the WHBP is the lack of anesthesia, which may explain the different respiratory effects observed in our study which used isoflurane anesthesia. Although blood gases were not measured, it is likely the mice were hypercapnic during our anesthetized experiments. Orexin usually stimulates breathing to mitigate respiratory acidosis (Williams and Burdakov, 2008; Dias et al., 2009), but in the presence of anesthesia, endogenous orexin release from the lateral hypothalamus may be reduced. Our results suggest that the KF does not play a role in orexin-mediated hypercapnic ventilatory responses since breathing frequency transiently decreased following ORXR stimulation in the KF.

The WHBP also requires the removal of all forebrain structures (Paton et al., 2022). Opposite results of local ORX-B signaling in the KF of anesthetized, adult mice in our study may be due to the presence of additional circuit elements that link arousal or sleep/wake state with the breathing control network. In addition, the injections could have targeted slightly different populations of neurons. In the rat WHBP, injections were fairly rostral/ventral and likely overlapped with the location of XII pre-motoneurons. In a mouse, the location of KF neurons that project to the ventrolateral medulla is skewed more rostrally (Karthik et al., 2022), and overlaps with our injection sites.

Another component of our study design to consider is the lack of antagonist injections, such as suvorexant (Cox et al., 2010; Sharma and Amane, 2014), to reverse the effects of orexin receptor activation. However, since both ORX-B and MDK 5220 are selective orexin receptor agonists with very different chemical structures, we believe that the chances of off target effects contributing to changes in respiratory output were low under these experimental conditions.

## Conclusion

Breathing is an essential motor behavior that must be seamlessly coordinated with a variety of sensory and motor behaviors performed during daily life. Neuromodulatory influences from brain regions such as the hypothalamus help integrate arousal or sleep/wake state with breathing behavior (Sakurai, 2007). The present work shows how orexinergic signaling in the pontine, respiratory-controlling KF area can significantly change breathing opening possibilities for future *in vivo* studies that define the hypothalamic-pontine circuits for arousal and sleep/wake context-dependent control of respiration.

## Data Availability

The raw data supporting the conclusion of this article will be made available by the authors, without undue reservation.

## References

[B1] Chamberlin N. L. Saper C. B. (1994). Topographic organization of respiratory responses to glutamate microstimulation of the parabrachial nucleus in the rat. J. Neurosci. 14 (11 I), 6500–6510. 10.1523/jneurosci.14-11-06500.1994 7965054 PMC6577246

[B2] Cohen M. I. (1971). Switching of the respiratory phases and evoked phrenic responses produced by rostral pontine electrical stimulation. J. Physiol. 217 (1), 133–158. 10.1113/jphysiol.1971.sp009563 5571915 PMC1331548

[B3] Cox C. D. Breslin M. J. Whitman D. B. Schreier J. D. McGaughey G. B. Bogusky M. J. (2010). Discovery of the dual orexin receptor antagonist [(7 R)-4-(5-chloro-1, 3- benzoxazol-2-yl)-7-methyl-1, 4-diazepan-1-yl] [5-methyl-2-(2 H -1, 2, 3-triazol-2-yl)phenyl]methanone (MK-4305) for the treatment of insomnia. J. Med. Chem. 53 (14), 5320–5332. 10.1021/jm100541c 20565075

[B4] Deng B. S. Nakamura A. Zhang W. Yanagisawa M. Fukuda Y. Kuwaki T. (2007). Contribution of orexin in hypercapnic chemoreflex: Evidence from genetic and pharmacological disruption and supplementation studies in mice. J. Appl. Physiol. 103 (5), 1772–1779. 10.1152/japplphysiol.00075.2007 17717124

[B5] Dias M. B. Li A. Nattie E. E. (2009). Antagonism of orexin receptor-1 in the retrotrapezoid nucleus inhibits the ventilatory response to hypercapnia predominantly in wakefulness. J. Physiol. 587 (9), 2059–2067. 10.1113/jphysiol.2008.168260 19273574 PMC2689343

[B6] Dutschmann M. Herbert H. (2006). The Kölliker-Fuse nucleus gates the postinspiratory phase of the respiratory cycle to control inspiratory off-switch and upper airway resistance in rat. Eur. J. Neurosci. 24 (4), 1071–1084. 10.1111/j.1460-9568.2006.04981.x 16930433

[B7] Dutschmann M. Kron M. Mörschel M. Gestreau C. (2007). Activation of Orexin B receptors in the pontine Kölliker-Fuse nucleus modulates pre-inspiratory hypoglossal motor activity in rat. Respir. Physiol. Neurobiol. 159 (2), 232–235. 10.1016/j.resp.2007.06.004 17652033

[B8] Fung S. J. Yamuy J. Sampogna S. Morales F. R. Chase M. H. (2001). Hypocretin (orexin) input to trigeminal and hypoglossal motoneurons in the cat: A double-labeling immunohistochemical study. Brain Res. 903 (1–2), 257–262. 10.1016/s0006-8993(01)02318-6 11382413

[B9] Greco M. A. Shiromani P. J. (2001). Hypocretin receptor protein and mRNA expression in the dorsolateral pons of rats. Brain Res. Mol. Brain Res. 88 (1–2), 176–182. 10.1016/s0169-328x(01)00039-0 11295245

[B10] Harris G. C. Aston-Jones G. (2006). Arousal and reward: A dichotomy in orexin function. Trends Neurosci. 29 (10), 571–577. 10.1016/j.tins.2006.08.002 16904760

[B11] Horvath T. L. Peyron C. Diano S. Ivanov A. Aston-Jones G. Kilduff T. S. (1999). Hypocretin (orexin) activation and synaptic innervation of the locus coeruleus noradrenergic system. J. Comp. Neurol. 415 (2), 145–159. 10.1002/(sici)1096-9861(19991213)415:2<145:aid-cne1>3.0.co;2-2 10545156

[B12] Irukayama-Tomobe Y. Ogawa Y. Tominaga H. Ishikawa Y. Hosokawa N. Ambai S. (2017). Nonpeptide orexin type-2 receptor agonist ameliorates narcolepsy-cataplexy symptoms in mouse models. Proc. Natl. Acad. Sci. U. S. A. 114 (22), 5731–5736. 10.1073/pnas.1700499114 28507129 PMC5465922

[B13] Ivanov A. Aston-Jones G. (2000). Hypocretin/orexin depolarizes and decreases potassium conductance in locus coeruleus neurons. Neuroreport 11 (8), 1755–1758. 10.1097/00001756-200006050-00031 10852238

[B14] Karthik S. Huang D. Delgado Y. Laing J. J. Peltekian L. Iverson G. N. (2022). Molecular ontology of the parabrachial nucleus. J. Comp. Neurol. 530 (10), 1658–1699. 10.1002/cne.25307 35134251 PMC9119955

[B15] Kaur S. Saper C. B. (2019). Neural circuitry underlying waking up to hypercapnia. Front. Neurosci. 13 (APR), 401. 10.3389/fnins.2019.00401 31080401 PMC6497806

[B16] Kilduff T. S. Peyron C. (2000). The hypocretin/orexin ligand-receptor system: Implications for sleep and sleep disorders. Trends Neurosci. 23, 359–365. 10.1016/s0166-2236(00)01594-0 10906799

[B17] Kuwaki T. (2008). Orexinergic modulation of breathing across vigilance states. Respir. Physiol. Neurobiol. 164 (1–2), 204–212. 10.1016/j.resp.2008.03.011 18455970

[B18] Kuwaki T. Zhang W. Nakamura A. Deng B. S. (2008). Emotional and state-dependent modification of cardiorespiratory function: Role of orexinergic neurons. Auton. Neurosci. 142, 11–16. 10.1016/j.autneu.2008.03.004 18440283

[B19] Lavezzi A. M. Ferrero S. Roncati L. Matturri L. Pusiol T. (2016). Impaired orexin receptor expression in the kölliker–fuse nucleus in sudden infant death syndrome: Possible involvement of this nucleus in arousal pathophysiology. Neurol. Res. 38 (8), 706–716. 10.1080/01616412.2016.1201632 27353953

[B20] Lazarenko R. M. Stornetta R. L. Bayliss D. A. Guyenet P. G. (2011). Orexin A activates retrotrapezoid neurons in mice. Respir. Physiol. Neurobiol. 175 (2), 283–287. 10.1016/j.resp.2010.12.003 21145990 PMC3032025

[B21] Lumsden T. (1923). Observations on the respiratory centres in the cat. J. Physiol. 57 (3–4), 153–160. 10.1113/jphysiol.1923.sp002052 16993609 PMC1405470

[B22] Lydic R. Orem J. (1979). Respiratory neurons of the pneumotaxic center during sleep and wakefulness. Neurosci. Lett. 15 (2–3), 187–192. 10.1016/0304-3940(79)96111-1 231237

[B23] Marcus J. N. Aschkenasi C. J. Lee C. E. Chemelli R. M. Saper C. B. Yanagisawa M. (2001). Differential expression of Orexin receptors 1 and 2 in the rat brain. J. Comp. Neurol. 435 (1), 6–25. 10.1002/cne.1190 11370008

[B24] Nakamura A. Zhang W. Yanagisawa M. Fukuda Y. Kuwaki T. (2007). Vigilance state-dependent attenuation of hypercapnic chemoreflex and exaggerated sleep apnea in orexin knockout mice. J. Appl. Physiol. 102 (1), 241–248. 10.1152/japplphysiol.00679.2006 16959906

[B25] Nambu T. Sakurai T. Mizukami K. Hosoya Y. Yanagisawa M. Goto K. (1999). Distribution of orexin neurons in the adult rat brain. Brain Res. 827 (1–2), 243–260. 10.1016/s0006-8993(99)01336-0 10320718

[B26] Nattie E. Li A. (2012). “Respiration and autonomic regulation and orexin,” in Progress in brain research, 25–46. 10.1016/B978-0-444-59489-1.00004-5 PMC4405125 22813968

[B27] Oliven R. Cohen G. Somri M. Schwartz A. R. Oliven A. (2020). Relationship between the activity of the genioglossus, other peri-pharyngeal muscles and flow mechanics during wakefulness and sleep in patients with OSA and healthy subjects. Respir. Physiol. Neurobiol. 274, 103362. 10.1016/j.resp.2019.103362 31866501 PMC7422939

[B28] Paton J. F. R. Machado B. H. Moraes D. J. A. Zoccal D. B. Abdala A. P. Smith J. C. (2022). Advancing respiratory-cardiovascular physiology with the working heart-brainstem preparation over 25 years. J. Physiol. 600 (9), 2049–2075. 10.1113/JP281953 35294064 PMC9322470

[B29] Peyron C. Tighe D. K. Van Den Pol A. N. De Lecea L. Heller H. C. Sutcliffe J. G. (1998). Neurons containing hypocretin (orexin) project to multiple neuronal systems. J. Neurosci. 18 (23), 9996–10015. 10.1523/jneurosci.18-23-09996.1998 9822755 PMC6793310

[B30] Remmers J. E. DeGroot W. J. Sauerland E. K. Anch A. M. (1978). Pathogenesis of upper airway occlusion during sleep. J. Appl. Physiol. Respir. Environ. Exerc. Physiol. 44 (6), 931–938. 10.1152/jappl.1978.44.6.931 670014

[B31] Sakurai T. Amemiya A. Ishii M. Matsuzaki I. Chemelli R. M. Tanaka H. (1998). Orexins and orexin receptors: A family of hypothalamic neuropeptides and G protein-coupled receptors that regulate feeding behavior. Cell 92 (4), 696–785. 10.1016/s0092-8674(02)09256-5 9491897

[B32] Sakurai T. (2007). The neural circuit of orexin (hypocretin): Maintaining sleep and wakefulness. Nat. Rev. Neurosci. 8 (3), 171–181. 10.1038/nrn2092 17299454

[B33] Schindelin J. Arganda-Carreras I. Frise E. Kaynig V. Longair M. Pietzsch T. (2012). Fiji: An open-source platform for biological-image analysis. Nat. Methods 9, 676–682. 10.1038/nmeth.2019 22743772 PMC3855844

[B34] Sharma S. Amane H. (2014). Suvorexant, novel dual orexin receptor antagonist for management of insomnia: A systematic review. Indian J. Pharmacol. 1, S106. Available from: http://ovidsp.ovid.com/ovidweb.cgi?T=JS&CSC=Y&NEWS=N&PAGE=fulltext&D=emed13&AN=72168430, http://sfx.ucl.ac.uk/sfx_local?sid=OVID:embase&id=pmid:&id=:&issn=0253-7613&isbn=&volume=46&issue=7+SUPPL.+1&spage=S106&pages=S106&date=2014&title=Indian+Journal+of+.

[B35] Sieck G. C. Harper R. M. (1980). Pneumotaxic area neuronal discharge during sleep-waking states in the cat. Exp. Neurol. 67 (1), 79–102. 10.1016/0014-4886(80)90162-4 7349988

[B36] Toyama S. Shimoyama N. Tagaito Y. Nagase H. Saitoh T. Yanagisawa M. (2018). Nonpeptide orexin-2 receptor agonist attenuates morphine-induced sedative effects in rats. Anesthesiology 128 (5), 992–1003. 10.1097/ALN.0000000000002161 29521652

[B37] Varga A. G. Maletz S. N. Bateman J. T. Reid B. T. Levitt E. S. (2020). Neurochemistry of the Kölliker-Fuse nucleus from a respiratory perspective. J. Neurochem. 156, 16–37. 10.1111/jnc.15041 32396650 PMC7657983

[B38] Wang C. Wang Q. Ji B. Pan Y. Xu C. Cheng B. (2018). The orexin/receptor system: Molecular mechanism and therapeutic potential for neurological diseases. Front. Mol. Neurosci. 11, 220. 10.3389/fnmol.2018.00220 30002617 PMC6031739

[B39] Williams R. H. Burdakov D. (2008). Hypothalamic orexins/hypocretins as regulators of breathing. Expert Rev. Mol. Med. 10 (28), e28–e10. 10.1017/S1462399408000823 18828950 PMC5767117

[B40] Yokota S. Oka T. Asano H. Yasui Y. (2016). Orexinergic fibers are in contact with Kölliker-Fuse nucleus neurons projecting to the respiration-related nuclei in the medulla oblongata and spinal cord of the rat. Brain Res. 1648, 512–523. 10.1016/j.brainres.2016.08.020 27544422

[B41] Young J. K. Wu M. Manaye K. F. Kc P. Allard J. S. Mack S. O. (2005). Orexin stimulates breathing via medullary and spinal pathways. J. Appl. Physiol. 98 (4), 1387–1395. 10.1152/japplphysiol.00914.2004 15557013

